# Effective accreditation in postgraduate medical education: from process to outcomes and back

**DOI:** 10.1186/s12909-020-02123-3

**Published:** 2020-09-28

**Authors:** Glen Bandiera, Jason Frank, Fedde Scheele, Jolanta Karpinski, Ingrid Philibert

**Affiliations:** 1 University of Toronto, Toronto, Canada; 2 Royal College of Physicians and Surgeons of Canada, Ottawa, Canada; 3 OLVG Teaching Hospital, Amsterdam, The Netherlands; 4 VU Medical Center, School of Medical Sciences, Amsterdam, The Netherlands; 5 Athena Institute for Transdisciplinary Research, Amsterdam, The Netherlands; 6 Department of Medical Education, Frank H. Netter MD School of Medicine at Quinnipiac University, North Haven, CT USA

**Keywords:** Accreditation, Competency frameworks, Clinical outcomes, Outcome measures, Process measures, Societal accountability

## Abstract

**Background:**

The accreditation of medical educational programs is thought to be important in supporting program improvement, ensuring the quality of the education, and promoting diversity, equity, and population health. It has long been recognized that accreditation systems will need to shift their focus from processes to outcomes, particularly those related to the end goals of medical education: the creation of broadly competent, confident professionals and the improvement of health for individuals and populations. An international group of experts in accreditation convened in 2013 to discuss this shift.

**Main text:**

Participants unequivocally supported the inclusion of more outcomes-based criteria in medical education accreditation, specifically those related to the societal accountability of the institutions in which the education occurs. Meaningful and feasible outcome metrics, however, are hard to identify. They are regionally variable, often temporally remote from the educational program, difficult to measure, and susceptible to confounding factors. The group identified the importance of health outcomes of the clinical milieu in which education takes place in influencing outcomes of its graduates. The ability to link clinical data with individual practice over time is becoming feasible with large repositories of assessment data linked to patient outcomes. This was seen as a key opportunity to provide more continuous oversight and monitoring of program impact. The discussants identified several risks that might arise should outcomes measures completely replace process issues. Some outcomes can be measured only by proxy process elements, and some learner experience issues may best be measured by such process elements: in brief, the “how” still matters.

**Conclusions:**

Accrediting bodies are beginning to view the use of practice outcome measures as an important step toward better continuous educational quality improvement. The use of outcomes will present challenges in data collection, aggregation, and interpretation. Large datasets that capture clinical outcomes, experience of care, and health system performance may enable the assessment of multiple dimensions of program quality, assure the public that the social contract is being upheld, and allow identification of exemplary programs such that all may improve. There remains a need to retain some focus on process, particularly those related to the learner experience.

## Background

The accreditation of physician education programs is meant to assure the medical community and the public of the quality of education and its relevance to medical practice. Accreditation is also a means of stimulating change in medical education, physician practice, and health care systems [1]. The formulation of accreditation standards for physician education has been driven by the need toimprove patient care and population health, reduce the cost of care [2], address learner expectations [3], and respond to concerns about equity in medical education [4, 5]. There is a growing interest in collecting and using outcomes data as part of the accreditation process; that is, using outcomes related to physicians’ professional practice after graduation as a metric for the effectiveness of educational programs [6, 7]. Frameworks for assessing educational outcomes related to the competencies of graduates include the CanMeds 2015 Physician Competency Framework (Royal College of Physician and Surgeons of Canada) [8]; the Milestones Project (US Accreditation Council for Graduate Medical Education) [9]; Tomorrow’s Doctor (UK General Medical Council) [10], and the Scottish Doctor (Scottish Deans’ Medical Education Group) [11]. Although these frameworks span undergraduate and postgraduate medical education and are tailored to the needs of health care systems in various countries, all share an interest in identifying the attributes of high-performing physicians [12, 13]. Concurrently, there is an interest in linking the accreditation of physician education programs to clinical outcomes, thus building on the limited existing evidence that accreditation benefits patients and health systems by producing physicians who are better prepared for practice [12–14]. This is an important objective in light of some misgivings that have surrounded the movement toward competency-based medical education (CBME) – which include the perception that CBME adheres to a reductionist framework that may not capture all dimensions of physician practice [3] and concerns that competency-based approaches may encourage a preoccupation with accelerating learning rather than ensuring that all learners are prepared for practice [15].

In the fall of 2013, the Royal College of Physicians and Surgeons of Canada convened an international group of accreditation experts and educators at a summit held in conjunction with the International Conference on Residency Education to discuss the future of postgraduate program accreditation. Working groups were charged with carrying out in-depth analyses of topics relevant to the accreditation of residency programs. This paper presents the deliberations of a working group that addressed educational process and outcomes in relation to the accreditation of residency programs. The group considered three questions: (1) What are accreditation outcomes? (2) What are the respective roles of process and outcome measures in accreditation? (3) What outcome measures can be used in the accreditation of postgraduate physician education programs? The one-day summit involved background sessions, a review of the literature, and round-table discussions. A subgroup distilled the findings into a report, which was vetted by participants and formed the foundation for this paper, which is focused on the roles of process measures and outcome measures in accreditation. We plan to discuss social accountability expectations for postgraduate medical education in a future publication [16].

## Main text

### Competence domains and educational and clinical outcomes in accreditation

In 1998 Donabedian proposed a model for the assessment of the quality of care in which “the information from which inferences can be drawn about the quality of care can be classified under three categories: ‘structure,’ ‘process,’ and ‘outcomes’” [17]. Similarly, accreditation criteria in medical education have long incorporated structural requirements (e.g., as related to faculty, clinical space, and technology) and process requirements (e.g., pertaining to procedural volume and length and type of clinical and didactic experiences) [1]. However, an emphasis on practice outcomes as a quality measure in the accreditation of medical programs has emerged only recently, in tandem with the shift toward CBME. CBME focuses on the knowledge, skills, and attributes of graduates of physician education programs and enhances accountability for learner development in these domains [1, 7, 13]. This increases accountability to stakeholders, including learners and those who will be served by the future graduates of accredited programs [1, 13]. Competency-based frameworks are similar across nations, as shown by the comparative summaries given in Table 1.

**Table 1:** Outcomes-focused accreditation dimensions in four national frameworks

CanMEDS Roles [8]	ACGME Competencies [9]	Tomorrow’s Doctor [10]	The Scottish Doctor [11]
Medical Expert	Medical Knowledge	Knowledge, skills and performance	Basic, social and clinical sciences and underlying principles
	Patient Care		Clinical skills
			Practical procedures
			Patient investigation
			Patient management
			Decision-making skills and clinical reasoning and judgement
Communicator	Interpersonal and Communication Skills	Communication, partnership and teamwork	Communication
			Medical informatics
Collaborator	Systems-Based Practice		The role of the doctor within the health service
Leader			
Scholar	Practice-Based Learning and Improvement		Personal development
Health Advocate		Safety and quality	Health promotion and disease prevention
Professional	Professionalism	Maintaining trust	Attitudes, ethical understanding and legal responsibilities

### Learning, patient care, and health systems outcomes

Outcomes can be categorized as learning outcomes; patient and population health outcomes; and health system outcomes. Our working group considered immediate learning outcomes, including performance on in-training written examinations, objective structured clinical examinations [18], graduation rates, and standardized certification examinations taken by graduates, along with surveys of graduates in practice and of the institutions that employ them. At present, other than performance on certification examinations, the use of educational outcomes to assess the impact of accreditation is largely non-existent. This may be due to the lack of availability of data related to practice patterns and patient outcomes, lack of consensus on meaningful measures that can be traced to educational programming, or resource constraints that prohibit programs and institutions for pursuing such activities with rigour. Many of these historic challenges may be mitigated by imminent evolutions in data stewardship discussed later in this paper. The focus group participants also identified outcomes at level of the patient and the health care system. Furthermore, it was felt that relevant outcomes may vary depending on the population and sociocultural milieu for which they are contemplated. Table 2 shows a framework of metrics that can be mapped to the competency expectations for physicians outlined in Table 1, across a sample dichotomous scale related to state of economic development. For example, communication skills are referenced in all four frameworks, and good communication skills have been associated with positive outcomes such as patient satisfaction and increased adherence to therapy [19]. We propose that, collectively, the outcome measures listed in Table 2 could be used as indicators of program effectiveness. The table shows that, beyond metrics that relate to the emerging postgraduate medical education enterprise in developing economies, and a greater focus on chronic and lifestyle-related conditions in developed economies, outcomes to assess the performance of medical education systems vary relatively little between the two types of economies.

**Table 2:** A sample working framework for accreditation based on patient care and health system outcomes

	Learning outcomes	Patient/patient care outcomes	Health system outcomes
Developed economies	Residency/fellowship completion rates In-training examination performance Licensing and certification examination performance (initial and re-examination) Surveys of program graduates	Ability to care for patients with a variety of common diagnoses and conditions Quality of care for groups of patients with acute, chronic, and lifestyle-related diseases and conditions Complication rates for procedures Patient-reported outcomes (patient experience of care)	Cost effectiveness in patient management Health care costs as a percentage of gross domestic product Patient access to care Population health indicators Potentially, practice location and scope of practice for graduates
Developing economies	Training program completion rates Physicians who received their graduate medical education locally Subspecialty physicians trained abroad who return to practise in the country Licensing and board examination performance Surveys of program graduates	Ability to care for patients with a variety of common diagnoses and conditions Quality of care for patients with acute and chronic conditions Complication rates for procedures Patient-reported outcomes (patient experience of care)	Cost-effectiveness in patient management Patient access to care Population health indicators Potentially, practice location and scope of practice for graduates

Research on the impact of education on outcomes in practice has made a number of links between educational outcomes and patient outcomes. For example, one study found that poor communication scores in licensure examination was predictive of a greater frequency of patient complaints to regulatory authorities [20], while other studies found an association between higher licensing examination scores and higher rates of consultation, better prescribing practices, higher mammography screening rates, and more appropriate prescribing for elderly patients, and also demonstrated the persistence of these effects [21, 22]. A large study of obstetrical patients showed an enduring association between the quality of clinical care in the residency program and the quality of care graduates deliver for a decade and a half into practice [23]. At the health system level, studies have shown an association between the characteristics of residency programs and graduates’ ability to practice conservatively [24]; another study found that a cost-conservative style of practice associated with residency programs in certain regions persisted 15 years after training [25].

One potential outcome of educational programs is the societal impact of the graduates, as determined by their clinical practice “footprint.” In the discussion of specialty, and location and scope of practice, participants agreed that programs should be held accountable for, and assessed against, only those outcomes within their control. The discussion emphasized that various factors influence graduates’ choice of specialty and practice location, including remuneration, health care resource allocation, incentive packages, and professional and lifestyle considerations. These factors may affect the ability of individual programs to produce physicians who represent the best fit for the health care needs of their area, region, or country. This raises the question as to whether it is appropriate to place responsibility for physician workforce considerations at the level of the individual program, or whether this responsibility should more appropriately be assumed by institutions or entities at the regional or national level. Participants emphasized that, at a minimum, postgraduate education programs should be active partners in health human resource planning and in educating learners about their obligations and opportunities to fulfill societal responsibilities. Accreditation bodies should assess the design and impact of programs’ attempts to address these societal obligations.

### Challenges in data collection and use

Collectively, the studies cited in the previous section show that the quality of teaching and patient care in settings where residents train has an impact on their future practice. Performance in practice as an outcome measure has been proposed as a key innovation in the accreditation systems of the future [26], but to date no accreditation frameworks have been able to incorporate practice outcomes into their assessment process. The reasons for this include (1) data collection and measurement challenges; (2) the inherent difficulty of attributing outcomes to specific individual practice let alone to their past education; and (3) the time lag between the completion of training and many outcomes of interest to accreditors.

The first challenge pertains to the burden and cost of data collection, which must be weighed against the strength of the impact of the clinical environment on graduates’ performance in practice. That is to say, is it worth the cost of data collection if the degree of correlation or potential for modification are poor? It is often difficult to identify feasible, meaningful measures for outcomes of interest. The existing studies used homogeneous patient populations, national billing data, and data links between certification and licensure authorities, but these do not exist for all relevant patient groups, or in all countries. In addition, there are questions about data ownership and use, and this area will require work to address privacy concerns and other legal considerations. The second challenge relates to the ability to unequivocally and fairly attribute educational outcomes to the unit of interest: the program. Educational attainment and performance in practice are influenced by individual abilities as well as by the characteristics of the education. A highly selected group of learners may perform well regardless of the effectiveness of their education program or its accreditation, while a high-acuity group of patients served by graduates may experience poorer outcomes regardless of the effectiveness of the education or accreditation system. The third challenge is that many outcomes do not become apparent until long after a cohort completes training; given this time lag, feedback may come too late for programs to make meaningful changes. This delay could put trainees at risk for substandard education if process measures were replaced *en bloc* by outcome measures.

### The respective roles of outcome and process measures

One theme that emerged from our discussions pertained to the relationship between educational processes and outcomes. Some participants advocated a focus on educational outcomes, noting that reducing the emphasis on adherence to process requirements would reduce the burden that the accreditation process places on educational programs and would also allow them greater freedom to innovate [1]. However, the majority of participants noted that, even in the absence of explicit compliance mandates, programs need to be attentive to process, including educational methods and attributes of the working and learning environment, given the multiple studies that have demonstrated the impact of these factors on performance in practice [20–25]. These participants felt that an exclusive focus on outcomes by accreditors could undervalue best practices in resident education, and affirmed their interest in retaining process dimensions that have been shown to be important from an educational, patient care, and learning-environment perspective. They also noted that interventions to improve quality will occur in various process dimensions, such as curricula, learning experiences, assessment, and ensuring a safe, supportive, and respectful learning environment. That being said, some process elements that serve as surrogates for outcomes could be replaced by more direct measures of program success. Similarly, antiquated and problematic indicators such as case counts and time on task can be retired in lieu of objective competency assessments.

## Recommendations

There was a consensus that the assessment of competency, both in training and in practice, provides useful data for the quality assessment of postgraduate medical education. At the same time, participants appreciated the inherent challenges in measuring outcomes and using this information to generate timely, actionable feedback to foster improvement. They also agreed that process measures remain critical to high-quality education and accreditation. The discussion resulted in a set of recommendations for the use of process and outcome measures in accreditation, organized by responsible entity.

### Recommendations for postgraduate education programs

- *Recommendation 1:* Data collected at the program level should assess whether a graduating physician is fully “practice ready,” that is, competent in the full spectrum of practice in his or her chosen field.
- *Recommendation 2:* All residents and fellow portfolios should have a career planning component, to help ensure that their final period of training addresses residual gaps and to enhance their own understanding of their readiness for practice.
- *Recommendation 3:* Through self-reflection, preceptor and mentor support, and program director review, trainees should periodically identify areas of strength and areas in need of further development.
- *Recommendation 4:* Programs should ensure that each graduate displays the characteristics and behaviours necessary to work effectively in interprofessional teams. Data to inform assessment should come from peers, faculty, and health professionals such as nurses, pharmacists, and others.
- *Recommendation 5:* Programs should report on their efforts to address residents with learning or professional challenges, and on remediation and development strategies they have found effective.
- *Recommendation 6:* Programs should collect some outcomes data, such as feedback from graduates, and use this information to improve and innovate educational processes.

### Recommendations at the accrediting organization level

- *Recommendation 7:* Accrediting organizations should identify outcome data that offer meaningful, “near-real-time” feedback for use by programs in ongoing improvement activities.
- *Recommendation 8:* Accrediting organizations should identify or develop process indicators that specifically allow oversight of learning environment issues.
- *Recommendation 9:* Accrediting organizations should develop and deploy an appropriate mix of standards pertaining to both process outcomes and patient care outcomes, to promote program and educational quality improvement, create value in accreditation, and meet societal needs.

## Conclusion

Accrediting bodies are beginning to focus on continuous educational quality improvement, and view the use of practice outcome measures as an important step forward. These approaches may take time to embed themselves in medical education, yet will result in improvements that otherwise may not be feasible. The use of outcomes in accreditation is a promising development, but it also presents challenges in data collection, aggregation, and interpretation; moreover, process and outcome measures will continue to be used collectively. This international group of experts and stakeholders highlights challenges with accreditation as a means for national health workforce planning, and identified the need for a continued dual focus on process and outcomes. Accreditors will need to design a system that uses these attributes in a meaningful, effective, and efficient way.

In the not-to-distant future, large datasets that capture clinical outcomes, experience of care, and health system performance may provide a rich constellation of information to assess multiple dimensions of program quality and assure the public that the social contract to train competent physicians is being upheld. An attractive feature is the potential to identify exemplary programs. For such exemplars, the focus will assuredly be on the processes that contribute to their superior outcomes.

## Data Availability

Not applicable.

## Abbreviation

CBME: Competency-based medical education

## References

[1] Nasca TJ, Philibert I, Brigham T, Flynn TC (2012). The next GME accreditation system—rationale and benefits. N Engl J Med.

[2] Berwick DM, Nolan TW, Whittington J (2008). The triple aim: care, health, and cost. Health Aff (Millwood).

[3] Hawkins RE, Welcher CM, Holmboe ES, Kirk LM, Norcini JJ, Simons KB (2015). Implementation of competency-based medical education: are we addressing the concerns and challenges?. Med Educ.

[4] Girotti JA, Park YS, Tekian A (2015). Ensuring a fair and equitable selection of students to serve society’s health care needs. Med Educ.

[5] Cardinal LJ, Maldonado M, Fried ED (2016). A national survey to evaluate graduate medical education in disparities and limited english proficiency: a report from the AAIM diversity and inclusion committee. Am J Med.

[6] Frank JR, Snell L, Englander R, Holmboe ES, ICBME Collaborators (2017). Implementing competency-based medical education: moving forward. Med Teach.

[7] Hodges BD (2010). A tea-steeping or i-doc model for medical education?. Acad Med.

[8] Frank JR, Danoff D (2007). The CanMEDS initiative: implementing an outcomes-based framework of physician competencies. Med Teach..

[9] Batalden P, Leach D, Swing S, Dreyfus H, Dreyfus S (2002). General competencies and accreditation in graduate medical education. Health Aff (Millwood)..

[10] Watmough SD, O’Sullivan H, Taylor DC (2010). Graduates from a reformed undergraduate medical curriculum based on Tomorrow’s doctors evaluate the effectiveness of their curriculum 6 years after graduation through interviews. BMC Med Educ.

[11] Simpson JG, Furnace J, Crosby J, Cumming AD, Evans PA, Friedman Ben David M (2002). The Scottish doctor—learning outcomes for the medical undergraduate in Scotland: a foundation for competent and reflective practitioners. Med Teach..

[12] Institute of Medicine (IOM). Graduate medical education that meets the nation’s health needs [report]. 2014 Jul 29. Available from: www.nationalacademies.org/hmd/Reports/2014/Graduate-Medical-Education-That-Meets-the-Nations-Health-Needs.aspx. Accessed March 3, 2018. 25340242

[13] Council on Graduate Medical Education (2007). Eighteenth report. New paradigms for physician training for improving access to health care.

[14] Norcini JJ, van Zanten M, McGaw B, Peterson PL, Baker E (2009). Overview of accreditation, certification, and licensure processes. International encyclopaedia of education.

[15] Lucey CR, Thibault GE, Ten Cate O (2018). Competency-based, time-variable education in the health professions: crossroads. Acad Med.

[16] Philibert I, Blouin D. Responsiveness to societal needs in postgraduate medical education: the role of accreditation. BMC Med Educ. 2020;18(Suppl 1). 10.1186/s12909-020-02125-1. 10.1186/s12909-020-02125-1 PMC7520978 32981520

[17] Donabedian A (1988). The quality of care: How can it be assessed?. JAMA.

[18] Harden RM, Gleeson FA (1979). Assessment of clinical competence using an objective structured clinical examination (OSCE). Med Educ.

[19] King A, Hoppe RB (2013). “Best practice” for patient-centered communication: a narrative review. J Grad Med Educ.

[20] Tamblyn R, Abrahamowicz M, Dauphinee D, Wenghofer E, Jacques A, Klass D (2007). Physician scores on a national clinical skills examination as predictors of complaints to medical regulatory authorities. JAMA..

[21] Tamblyn R, Abrahamowicz M, Brailovsky C, Grand’Maison P, Lescop J, Norcini J (1998). Association between licensing examination scores and resource use and quality of care in primary care practice. JAMA..

[22] Tamblyn R, Abrahamowicz M, Dauphinee WD, Hanley JA, Norcini J, Girard N (2002). Association between licensure examination scores and practice in primary care. JAMA..

[23] Asch DA, Nicholson S, Srinivas S, Herrin J, Epstein AJ (2009). Evaluating obstetrical residency programs using patient outcomes. JAMA..

[24] Sirovich B, Lipner R, Johnston M, Holmboe E (2014). The association between residency training and internists’ ability to practice conservatively. JAMA..

[25] Chen C, Petterson S, Phillips R, Bazemore A, Mullan F (2014). Spending patterns in region of residency training and subsequent expenditures for care provided by practicing physicians for Medicare beneficiaries. JAMA..

[26] Nasca TJ, Weiss KB, Bagian JP, Brigham TP (2014). The accreditation system after the “Next Accreditation System”. Acad Med.

